# Diversity of the Morphometric and Biochemical Traits of *Allium cepa* L. Varieties

**DOI:** 10.3390/plants13131727

**Published:** 2024-06-22

**Authors:** Donata Arena, Hajer Ben Ammar, Nikola Major, Tvrtko Karlo Kovačević, Smiljana Goreta Ban, Nicolas Al Achkar, Giulio Flavio Rizzo, Ferdinando Branca

**Affiliations:** 1 Department of Agriculture, Food and Environment (Di3A), University of Catania, Via Valdisavoia 5, 95123 Catania, Italy; donata.arena@phd.unict.it (D.A.); hejer.biologie@gmail.com (H.B.A.); nicolas.alachkar@phd.unict.it (N.A.A.); giulio.rizzo@phd.unict.it (G.F.R.); fbranca@unict.it (F.B.); 2 Institute of Agriculture and Tourism, 52440 Poreč, Croatia; tvrtko@iptpo.hr (T.K.K.); smilja@iptpo.hr (S.G.B.)

**Keywords:** Egyptian walking onion, onion, shallot, antioxidant capacities, flavonoid content, phenolic compounds, sugars

## Abstract

Several *Allium cepa* L. varieties, representing a versatile set of vegetables widely utilized by consumers, are appreciated for their bioactive properties, including antimicrobial, anticarcinogenic, and antioxidant capacities. The aim of this study is to compare the morphometric characteristics and biochemical profiles of four cultivars of *A. cepa*, two of them represented by the perennial Sicilian landrace “Cipudda agghiarola” (*Allium* × *proliferum* (Moench) Schrader), widely known as the Egyptian walking onion (WO), and by the landrace “Cipudduzza” belonging to the variety known as *aggregatum* (ON), which were compared with two commercial cultivars of *A. cepa* var. *cepa* (onion), Stoccarda (OS) and Rossa Carmen (OR). The experimental trial was conducted in Catania (Sicily), following organic growing practices. The randomized complete block experimental design was adopted with one experimental factor, the genotype (GE) effect. The harvested plants were characterized for their main morphometric parameters, according to the International Plant Genetic Resources (IGPR) descriptors. The biochemical activity was assessed by analyzing the total phenolic content (TPC) and the total flavonoid content (TFC). The antioxidant capacity was determined by 2,2-diphenyl-1-picrylhydrazyl (DPPH) radical scavenging, ferric reducing antioxidant power (FRAP), and oxygen radical absorbance capacity (ORAC). The sugar profile (total sugars, sucrose, glucose, fructose, and fructooligosaccharides—FOS) and the volatile compounds by headspace-gas chromatography/mass spectrometry (HS-GC/MS) were also determined. The OR bulb exhibited the highest TPC (16.3 mg GAE/g d.w., *p* < 0.01) and TFC (8.5 mg QE/g d.w., *p* < 0.01), with the highest antioxidant capacity measured by the FRAP (27.1 µmol TE/g d.w., *p* < 0.01) and DPPH assays (46.2 µmol TE/g d.w., *p* < 0.01). The ON bulb showed the highest ORAC value (209 µmol TE/g d.w., *p* < 0.01). Generally, the bulbs were richer in sugars (584 mg/g d.w., *p* < 0.01) than the leaf blade (239 mg/g d.w., *p* < 0.01), except for OR. Significant interaction between the genotype and plant organ was noted in the volatile compound profiles (*p* < 0.05) except for total ketones and carboxylic acids, where higher content was observed in the leaf blade compared to the bulb, regardless of the genotype. These findings highlight WO’s potential for use in ready-to-eat products, enhancing its market value.

## 1. Introduction

The *Alliaceae* family comprises the *Allium* genus, rich in diversity and distinguished by significant morphological and phenotypical characteristics, encompassing both wild and cultivated types [1]. Onion (*Allium cepa* L.) is one of the most ancient bulb vegetables worldwide; it is a main ingredient in all cuisines and a valuable raw material in the food industry, and also plays an important role in regional food security. Until now, no alternative was able to replace it, due to its low production cost and its culinary versatility and nutritional composition [2,3]. *Allium cepa* L., originally domesticated in Central Asia (specifically in Iran and the Pakistan region), has been under cultivation for more than 5000 years. It is cultivated for both its dry bulbs and green leaves, with more than 110 million tons of dry bulbs and around 5 million tons of green onion every year, occupying more than 6 million ha and 230,000 ha for dry bulbs and green production, respectively, all around the world [4]. The significance of the differences between the different plant parts of the onion, such as bulbs and leaves, cannot be overstated. The leaves, visible above the soil surface, encompass the green shoots and leaves necessary for photosynthesis and subsequent energy production through solar radiation absorption [5]. Conversely, the bulbs, residing beneath the soil’s surface, act as a vital storage reservoir and accumulate nutrients important for the onion’s growth and metabolic processes [6]. Onion is a versatile vegetable utilized in numerous dishes, from curries to salads, serving as a condiment or blending with other vegetables, whether boiled or baked. It can be processed into different types of products, such as pickles, powders, pastes, and flakes [7,8]. *Allium cepa* L. is renowned for forming well-defined bulbs composed of inner fleshy leaves, exhibiting various shapes ranging from round to flattened globes or cylinders, and presenting different colors, including white, yellow, and red [9]. Several studies have shown that onions are a good source of flavonoids, particularly quercetin and its derivatives [10,11], as well as vitamins and minerals, including vitamins C and B6, folate, and potassium [12]. Quercetin, a flavonoid found in a variety of vegetables, is well-known for its antioxidant properties, protecting against free radicals, with preventive effects against cardiovascular diseases and anti-inflammatory actions [13,14,15]. Flavonols and anthocyanins are the main compounds found in red onions [16]. In addition, onions are rich in other bioactive compounds like fructooligosaccharides (FOS) and organosulfur compounds such as allyl-sulfides and thiosulfonates, which are responsible for the distinctive pungent flavor [17]. The enzyme alliinase converts the precursors of the sulfoxide compounds derived from S-Alk(en)yl-L-cysteine into allicin, a compound that is closely associated with the flavor and aroma of onions [18]. This reaction leads to the release of various compounds, including S-thiopropanal oxide, which induces the lacrimatory effect, pyruvic acid, and ammonia. Pyruvic acid, typically, is used as an indicator to define the level of pungency in onions [19]. In particular, allicin, methiin, propiin, iso-alliin, diallyl sulfide, and diallyl disulfide are the main compounds found in onions. Allicin exhibits antimicrobial and antioxidant properties, making it a subject of scientific interest for potential pharmaceutical and nutraceutical applications [20,21]. Furthermore, disulfide radicals have been identified in red onions [22]. The most abundant sulfur compounds are represented by di- and trisulfides, such as cis- and trans-methyl-1-propenyldisulfide, methyl-2-propenyl disulfide, dipropyl disulfide, cis- and trans-propenyl propyl disulfide, methyl propyl trisulfide, and dipropyl trisulfide [23]. Over the years, high diversity in onions was recorded, especially in the different regions of production; now, numerous cultivars are recognized as endogenous in their area of production. Within the species *Allium cepa* L., there exist numerous botanical varieties, such as the Egyptian walking onion. It was formerly classified as *Allium cepa* var. *viviparum* (Metzger); however, in 1983, Schubert et al. classified it as *Allium* × *proliferum*, originating from a hybridization between *A. cepa* and *A. fistulosum* (2n = 2x = 16), exhibiting intermediate morphological characteristics [2,24]. The Egyptian walking onion is a perennial neglected crop that is widespread in Sicily and is distinguished by its inflorescence, which is typically characterized by bulbils and sterile flowers or only bulbils [25]. Its propagation is vegetative as it does not produce seeds. The denomination of walking onion derives from the fact that due to the force of gravity, the shoots carrying the bulblets (4 to 12 bulblets) fall over onto the soil and induce new plant production away from the initial plant base at generally the length of the shoot, which can be up to 40 cm [26]. The bulblets, in general, can be planted at the end of August, with transplantation in autumn. The plants can be collected year-round except during May and August, which is the bulbification period. Numerous studies have highlighted the diversification of the Egyptian walking onion and its distinctive antioxidant profile, characterized by polyphenolic compounds, particularly flavonols (quercetin and its derivatives) and flavones such as apigenin and luteolin [27].

The main purpose of this study is to evaluate the biodiversity of *Allium cepa* L. varieties, comparing the perennial Sicilian landrace, the “Cipudda agghiarola” (Egyptian walking onion), and the landrace “Cipudduzza” (shallot) with two commercial cultivars of onion, Stoccarda and Rossa Carmen. This study will promote and reevaluate the Sicilian *Allium* landraces by highlighting their biodiversity, morphometric characteristics, and biochemical profiles.

## 2. Results

### 2.1. Morphometric Traits

Significant variations were observed for the morphometric parameters studied (Table 1). The characteristics of the plants were significantly affected by the genotype, except for leaf density, where no significant difference was observed between the investigated genotypes. BFW was highest in OR while the highest LBFW was observed in WO (28.0 and 27.1 g, respectively) compared to other investigated genotypes, except for OS (21.9 g). MLL, MLW, LA, and LC were also highest in WO compared to the other genotypes, with values of 48.1 cm, 10.4 cm, 6.8, and 6.4, respectively, except for MLL and LC, which were comparable to OS. Among the investigated genotypes, NL was highest in OS (9.0).

### 2.2. Biochemical Measurements

A significant interaction between the genotype and the plant organ (PO × GE) was observed (Table 2). The total phenolic content (TPC), total flavonoid content (TFC), antioxidant capacity (FRAP, DPPH, and ORAC), and sugar profile varied significantly in relation to the two experimental factors considered. The ANOVA results provide significant insights into the factors that affected the different parameters evaluated. 

#### 2.2.1. Total Phenolic Content (TPC)

**Figure 1:**
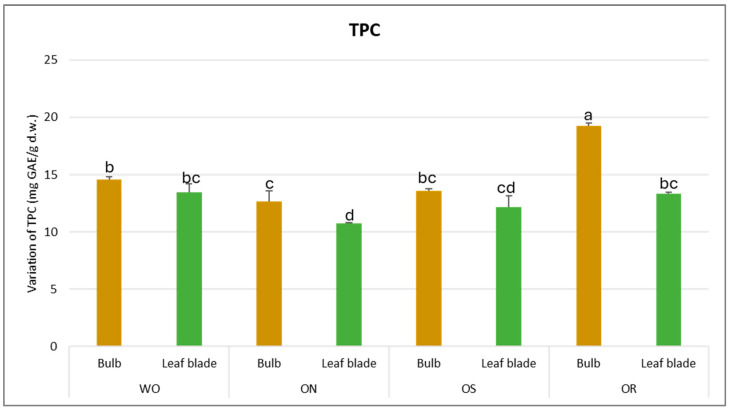
Variations in TPC (mg GAE/g d.w.) in the studied genotypes. Data are reported as mean ± standard error (n = 3). Different letters indicate significant differences according to the Tukey test (*p* ≤ 0.05). WO = Egyptian walking onion; ON = shallot; OS = Stoccarda; OR = Rossa Carmen.

Concerning the total phenolic content (TPC), a significant interaction in PO × GE was observed, with values ranging from 10.7 to 19.3 mg GAE/g d.w. between the ON leaf blade and OR bulb, respectively (Figure 1). A significantly higher TPC was observed for the OR bulb and ON leaf blade, while for WO and OS, there were no significant differences between the plant parts. Conversely, the OR bulb had the highest TPC content while the ON leaf blade had significantly lower TPC content compared to the WO and OS genotypes.

#### 2.2.2. Total Flavonoid Content (TFC)

**Figure 2:**
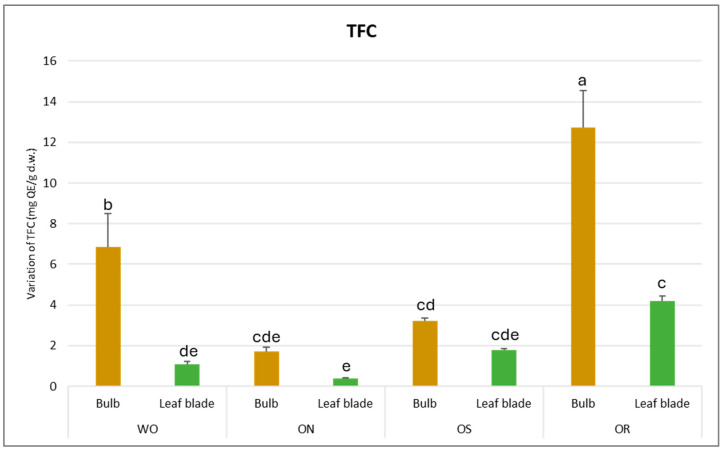
Variations in TFC (mg QE/g d.w.) in the studied genotypes. Data are reported as mean ± standard error (n = 3). Different letters indicate significant differences according to the Tukey test (*p* ≤ 0.05). WO = Egyptian walking onion; ON = shallot; OS = Stoccarda; OR = Rossa Carmen.

The total flavonoid content (TFC) was found to be significantly affected by both the plant organ and genotype, as outlined in Table 2. Notably, a significant interaction between plant organ and genotype (PO × GE) was observed, with TFC values varying from 12.7 mg of quercetin equivalents (QE)/g d.w. in the bulb of the OR genotype to 0.4 mg QE/g d.w. in the leaf blade (Figure 2). This demonstrates a remarkable disparity in TFC across different plant organs and genotypes. Specifically, the bulb, particularly in the OR and WO genotypes, exhibited the highest TFC compared to the leaf blade. Conversely, the ON genotype displayed the lowest TFC value, standing at 1.7 mg QE/g d.w.

#### 2.2.3. Total Antioxidant Capacity

**Figure 3:**
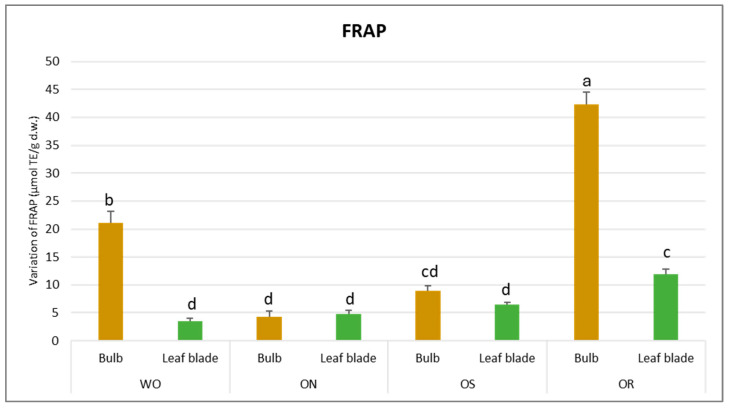
Variations in FRAP values (µmol TE/g d.w.) in the studied genotypes. Data are reported as mean ± standard error (n = 3). Different letters indicate significant differences according to the Tukey test (*p* ≤ 0.05). WO = Egyptian walking onion; ON = shallot; OS = Stoccarda; OR = Rossa Carmen.

The total antioxidant capacity was influenced by both the plant organ and genotype (Table 2). Significant variations were observed for FRAP activities, indicating a notable interaction between plant organs and genotype (PO × GE). Values ranged from 42.3 to 3.5 µmol TE/g d.w. for the OR bulb and WO leaf blade, respectively (Figure 3). 

**Figure 4:**
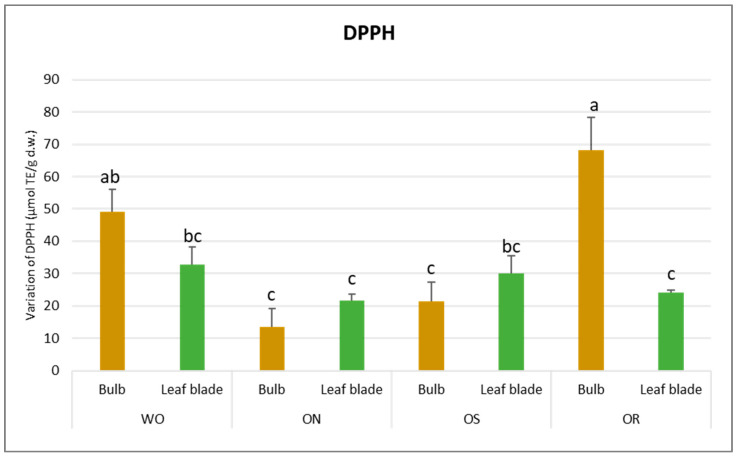
Variations in DPPH (µmol TE/g d.w.) in the studied genotypes. Data are reported as mean ± standard error (n = 3). Different letters indicate significant differences according to the Tukey test (*p* ≤ 0.05). WO = Egyptian walking onion; ON = shallot; OS = Stoccarda; OR = Rossa Carmen.

**Figure 5:**
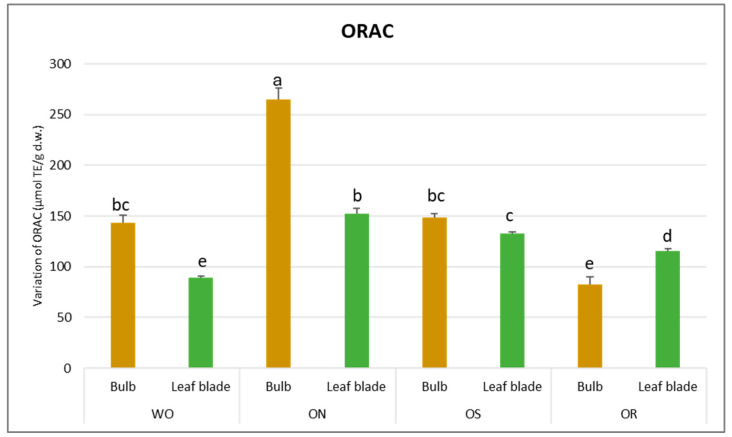
Variations in ORAC (µmol TE/g d.w.) in the studied genotypes. Data are reported as mean ± standard error (n = 3). Different letters indicate significant differences according to the Tukey test (*p* ≤ 0.05). WO = Egyptian walking onion; ON = shallot; OS = Stoccarda; OR = Rossa Carmen.

Regarding DPPH scavenging activity, a significant interaction of PO × GE was evident, with values ranging from 68.3 to 13.5 µmol TE/g d.w. for the OR bulb and ON bulb, respectively (Figure 4). The DPPH value of the WO bulb closely resembled that of the OR bulb, measuring 49.1 µmol TE/g d.w. Additionally, a significant interaction of plant organ and genotype (PO × GE) was noted for ORAC capacity. Values varied notably, ranging from 265 to 82.9 µmol TE/g d.w. for the ON bulb and OR bulb, respectively (Figure 5). Both the OS and WO bulbs exhibited notably high values, ranging from 149 to 144 µmol TE/g d.w., respectively. For both the DPPH radical scavenging and FRAP assay, the highest antioxidant capacity was detected in the OR bulb followed by the WO genotype, while the OS and ON had the lowest antioxidant capacity, as measured by the two tests. For the leaf blade, the OR had the highest FRAP values compared to other genotypes while in the DPPH radical scavenging assay, there were no significant differences between the investigated genotypes. The antioxidant capacity measured by FRAP was significantly higher in the bulbs compared to the leaf blades in WO and OR genotypes. The DPPH radical scavenging assay only showed higher antioxidant capacity for the bulb compared to the leaf blade in the OR genotype. The highest ORAC value was detected in the ON bulb, followed by OS and WO, and the lowest was detected in the OR bulb. For the leaf blade, the highest ORAC value was also obtained for ON, followed by OS, OR, and finally WO. The ON and WO bulbs had significantly higher ORAC values compared to the leaf blades while the opposite result was detected in the OR genotype.

#### 2.2.4. Soluble Sugar Analysis

**Figure 6:**
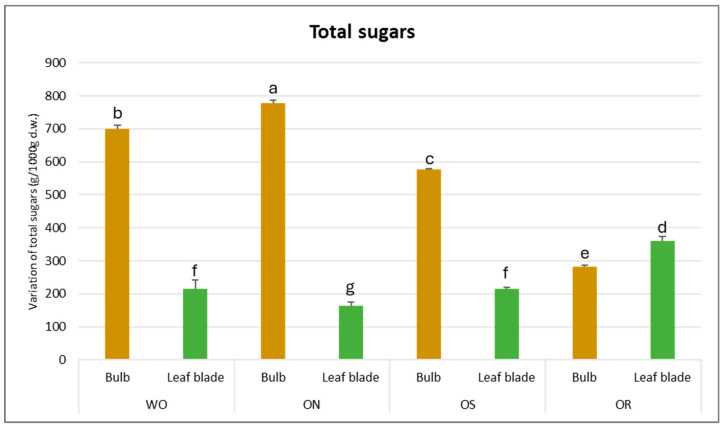
Variations in total sugars (g/kg d.w.) in the studied genotypes. Data are reported as mean ± standard error (n = 3). Different letters indicate significant differences according to the Tukey test (*p* ≤ 0.05). WO = Egyptian walking onion; ON = shallot; OS = Stoccarda; OR = Rossa Carmen.

The total sugar amount was significantly affected by the interaction between the plant organ and the genotype (PO × GE), with values ranging from 778 to 164 g/kg d.w. for the ON bulb and ON leaf blade, respectively (Figure 6). The highest content of total soluble sugars was determined for the bulbs compared to the leaf blades, except for OR, in which the value was highest for the leaf blades compared to the bulbs (361 g/kg d.w. and 282 g/kg d.w., respectively). The ON bulb showed the highest total sugar content, followed by WO and OS, while OR showed the lowest amount. For the leaf blade, the OR exhibited the highest value compared to the other genotypes.

**Figure 7:**
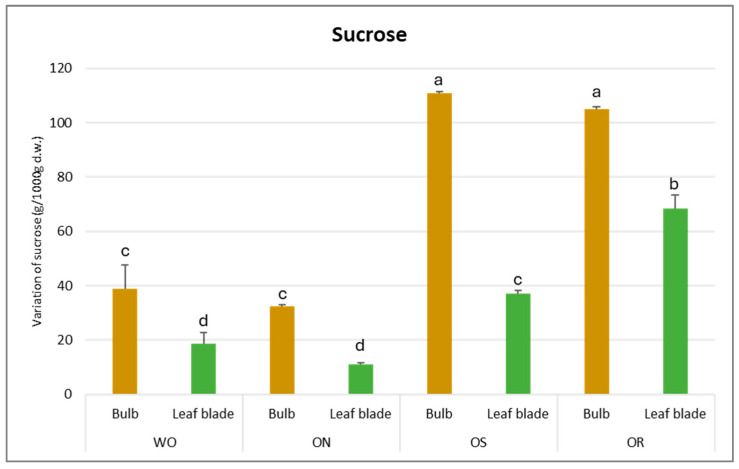
Variations in sucrose (g/kg d.w.) in the studied genotypes. Data are reported as mean ± standard error (n = 3). Different letters indicate significant differences according to the Tukey test (*p* ≤ 0.05). WO = Egyptian walking onion; ON = shallot; OS = Stoccarda; OR = Rossa Carmen.

The sucrose amount was significantly affected by PO and GE and a significant interaction between PO × GE was ascertained, with values varying from 111 to 11 g/kg d.w. for the OS bulb and ON leaf blade, respectively (Figure 7). The highest sucrose content was detected in the OS bulb, followed by OR and WO, and the lowest was detected in the ON bulb. For the leaf blade, the OR is highlighted as having the highest sucrose amount, followed by OS, while WO and ON showed the lowest sucrose content.

**Figure 8:**
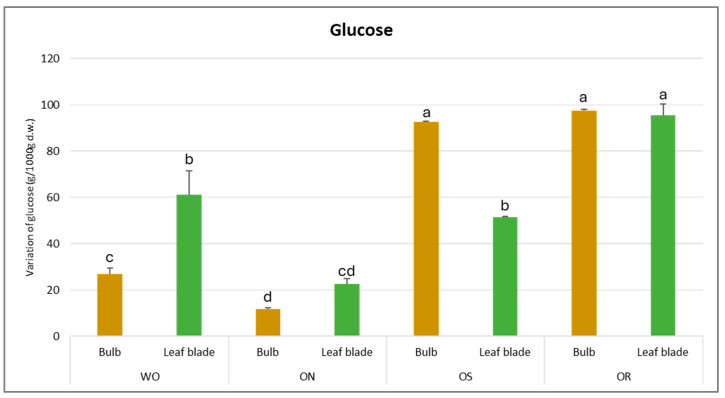
Variations in glucose (g/kg d.w.) in the studied genotypes. Data are reported as mean ± standard error (n = 3). Different letters indicate significant differences according to the Tukey test (*p* ≤ 0.05). WO = Egyptian walking onion; ON = shallot; OS = Stoccarda; OR = Rossa Carmen.

For glucose content, the results demonstrate a significant interaction of PO × GE and its value varied from 97.3 to 11.7 g/kg d.w. for the OR bulb and ON leaf blade, respectively (Figure 8). The highest glucose amount was ascertained for OR and OS bulbs, with values of 97.3 and 92.5 g/kg d.w., respectively. For the leaf blade, the highest glucose content was also detected in OR (95.6 g/kg d.w.), followed by the WO and OS genotypes, with values of 61.3 and 51.4 g/kg d.w., respectively. OR showed significantly higher glucose values in both the bulb and leaf blade compared to the other investigated genotypes, except for the OS bulb.

**Figure 9:**
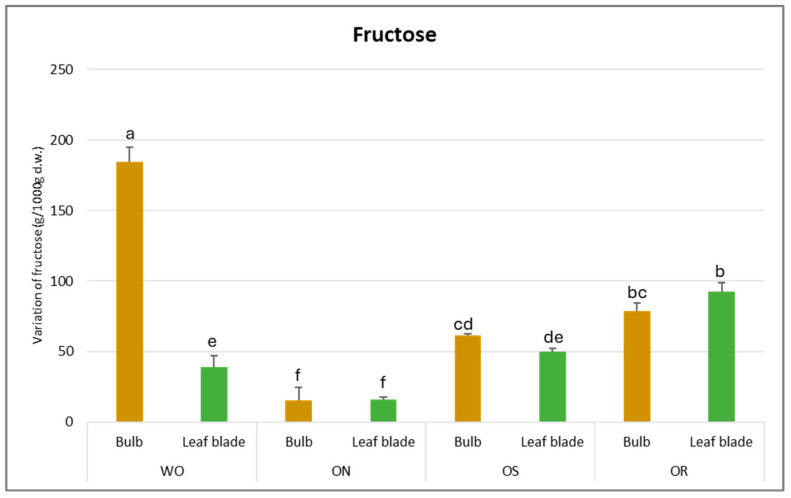
Variation of fructose (g/kg d.w.) in the studied genotypes. Data are reported as mean ± standard error (n = 3). Different letters indicate significant differences according to the Tukey test (*p* ≤ 0.05). WO = Egyptian walking onion; ON = shallot; OS = Stoccarda; OR = Rossa Carmen.

The fructose value varied from 184 g/kg d.w. to 16 g/kg d.w. for the WO bulb and the ON leaf blade, respectively (Figure 9). For the bulb, WO showed the highest fructose content, which was more than three times higher than the fructose content determined in the other genotypes. Conversely, for the leaf blade, OR exhibited the highest fructose value (92.3 g/kg d.w.). In general, the genotypes did not differ in fructose content between the plant parts except for WO, where a significantly higher fructose content in the bulb was observed compared to the leaf blade.

**Figure 10:**
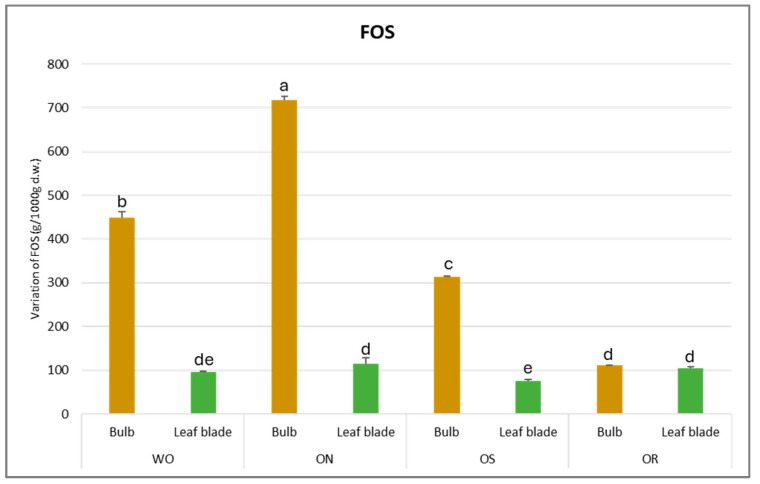
Variations in FOS (g/kg d.w.) in the studied genotypes. Data are reported as mean ± standard error (n = 3). Different letters indicate significant differences according to the Tukey test (*p* ≤ 0.05). WO = Egyptian walking onion; ON = shallot; OS = Stoccarda; OR = Rossa Carmen.

The fructooligosaccharide (FOS) values fluctuated from 718 to 76.0 g/kg d.w. for the ON bulb and OS leaf blade, respectively (Figure 10). For the bulb, the highest FOS content was observed in ON, followed by WO (449 g/kg d.w.), OS (313 g/kg d.w.), and finally, OR (104 g/kg d.w.). The OS genotype had significantly lower FOS content (76.0 g/kg d.w.) in the leaf blade compared to the OR and ON, comparable to that of WO. In general, the highest FOS amount was detected in the bulb for all the genotypes studied.

#### 2.2.5. Volatile Compounds Analysis

**Figure 11:**
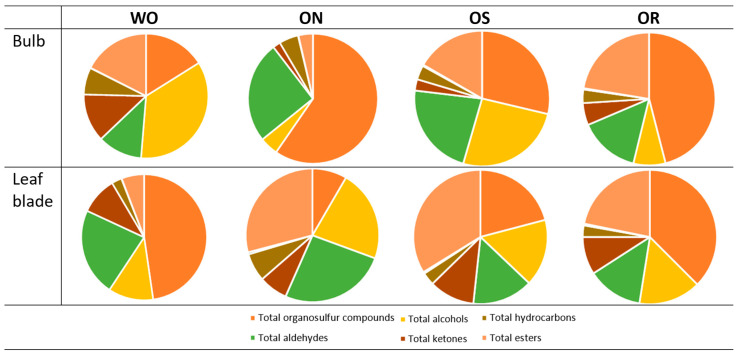
Percentage of volatile compounds in the profile. WO = Egyptian walking onion; ON = shallot; OS = Stoccarda; OR = Rossa Carmen.

The ANOVA of volatile compounds showed significant interactions between the plant organs and the investigated genotypes in all volatile groups except for the total ketones and total carboxylic acids (Table 3). The detailed volatile compound list detected in the bulbs and leaf blade of the investigated onion genotypes, as well as the ANOVA of each compound, is shown in Supplementary Table S1. The organosulfur compounds were the most abundant compounds found in the bulb and leaf blade. They represented 59.6% of the total volatile compounds in the ON bulb, followed by 47.7% in the WO bulb. In the leaf blade, the values ranged from 47.7 to 37.5% for WO and OR, respectively (Figure 11). Organosulfur compounds in the bulbs were highest in the ON genotype and the lowest in the WO genotype, while the opposite was observed in the leaf blade (Table 3). The ON and OS genotypes had significantly higher total organosulfur compound content in the bulb compared to the leaf blade, while the opposite was observed in the WO genotype. OS showed comparable total organosulfur compound content between bulbs and leaf blades. The main organosulfur compounds detected in both bulbs and leaf blades were organic sulfides, disulfides, and trisulfides, which are characteristic of allium plants (Supplementary Table S1). In the bulbs, alcohols were the second most abundant compounds, with values ranging from 35.2 to 25.8% for WO and OS, and leaf blade values fluctuating from 22.2 to 16.2% for ON and OS, respectively (Figure 11). Significantly higher total alcohols in were observed in the WO bulbs, followed by OS, compared to the ON and OR, while the ON genotype had the highest total alcohols in the leaf blade compared to the other investigated genotypes (Table 3). The main alcohols found in both bulbs and leaf blade of the investigated genotypes were 1-octen-3-ol and 1-nonanol, except for WO, where the predominant alcohol found was eugenol (Supplementary Table S1). Aldehydes were also highly represented volatile compounds in the tested genotypes, with percentage values ranging from 25.4 to 22.4% in the bulb for ON and OS, and from 26 to 22.6% in the leaf blade for ON and WO, respectively (Figure 11). The highest total aldehyde content was observed in the WO and ON leaf blades and was comparable to the ON bulb aldehyde content (Table 3). The total aldehydes were lowest in both the OR bulb and leaf blade, as well as in the WO bulb and OS leaf blade. The main aldehyde detected in both bulbs and leaf blades was 2-methy2-pentenal, except for the WO bulb and the ON leaf blade, where 2-undecenal and 3-methylbenzaldehyde were the main compounds, respectively. The onion leaf blade showed both higher total ketone and carboxylic acid contents compared to the bulb in all genotypes. The highest total ketones were observed in WO, while the lowest levels were found in ON. Total carboxylic acids were significantly higher in ON, OR, and OS compared to WO, regardless of plant organ. The main ketone observed in the leaf blades of OR, OS, and WO was 6,10-dimethyl-5,9-undecadien-2-one, while 2-tridecanone was the main ketone found in ON leaf blades (Supplementary Table S1). The main carboxylic acid found in both bulbs and leaf blades of the investigated onion genotypes was nonanoic acid (Supplementary Table S1). The highest bulb total hydrocarbon content was observed in WO, followed by ON, and the lowest was seen in the OR and OS bulbs. The highest level of leaf blade hydrocarbon content was detected in the ON compared to the other investigated genotypes and was comparable to WO bulb total hydrocarbon content. The percentage of esters in the bulb ranged from 22.4 to 17.5% in the OR and WO genotype, and from 33.8 to 29.1% in OS and ON, respectively (Figure 11). The highest leaf blade total ester content was observed in ON, followed by OS, and was significantly higher compared to both ON and OS bulb total ester content (Table 3). Conversely, WO had higher leaf blade total ester content compared to the bulb, while in the OR genotype, no significant difference was observed in the total ester content between the plant organs (Table 3). The main ester detected in both bulbs and leaf blades of all genotypes was ethyl heptanoate (Supplementary Table S1).

### 2.3. Correlation and Principal Component Analysis (PCA)

**Figure 12:**
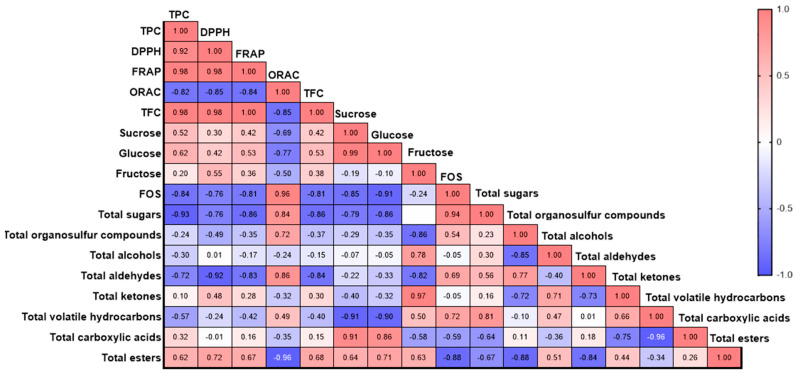
Pearson’s correlation coefficients among the different parameters studied.

Pearson correlation analysis showed different associations among the antioxidant profile, the sugar profile and content, and the profile of volatile compounds for the tested genotypes (Figure 12). The TPC was positively correlated with the antioxidant capacity measured by DPPH (r = 0.92) and FRAP (r = 0.98), while it was negatively correlated with the ORAC assay (r = −0.82). The TFC was significantly correlated with the TPC and the antioxidant capacity, with values of r = 0.98, r = 0.98, and r = 1.00 for TPC, DPPH, and FRAP, respectively, while a negative correlation was observed with ORAC (r = −0.85). A strong positive correlation was found between the soluble sugar content and the antioxidant capacity analyzed by different assays (Figure 12). Specifically, sucrose was positively correlated with TPC (r = 0.52), DPPH (r = 0.30), and FRAP (r = 0.42). Fructose also showed a positive correlation with the total phenolic content and the antioxidant capacities: TPC (r = 0.62), DPPH (r = 0.42), and FRAP (r = 0.53). However, sucrose, glucose, and fructose showed a negative correlation with ORAC. Additionally, a positive correlation was observed between the soluble sugars and the TFC, with r = 0.42, r = 0.53, and r = 0.38 for sucrose, glucose, and fructose, respectively. The FOS were negatively correlated with sucrose (r = −0.85), glucose (r = −0.91), and fructose (r = −0.24), as well as with TPC and antioxidant capacity, showing the following values: TPC (r = −0.84), DPPH (r = −0.76), and FRAP (r = −0.81). Moreover, FOS exhibited a positive correlation with ORAC (r = 0.96).

For volatile compounds, the organosulfur compounds showed a positive correlation with ORAC (r = 0.72) and FOS (r = 0.54). Fructose content was positively correlated with alcohols and ketones, with values of r = −0.78 and r = −0.97, respectively. Notably, the antioxidant capacity was negatively correlated with the majority of the volatile compounds registered, except for esters and carboxylic acids. Ester compounds were positively correlated with DPPH (r = 0.72) and FRAP (r = 0.67), while a negative correlation was observed with ORAC (r = −0.96). However, esters showed a positive correlation with TPC (r = 0.62) and sugars: sucrose (r = 0.64), glucose (r = 0.71), and fructose (r = 0.63).

**Figure 13:**
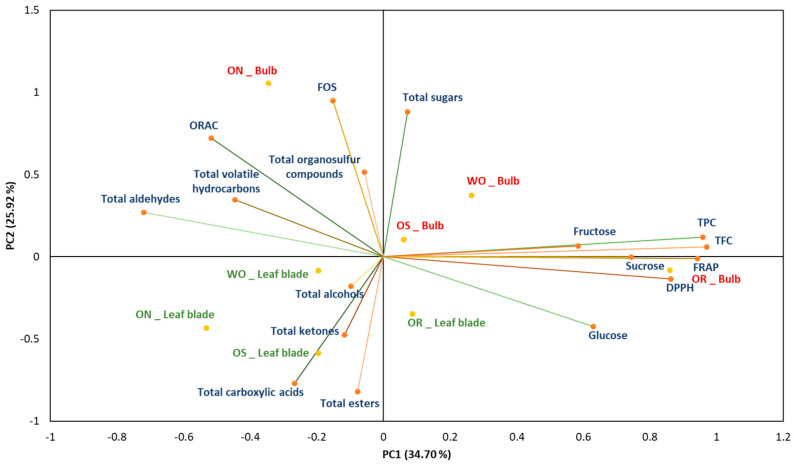
Principal component analysis biplot, with WO = Egyptian walking onion; ON = shallot; OS = Stoccarda; OR = Rossa Carmen. TPC = total phenolic content; TFC = total flavonoid content; FRAP = ferric reducing antioxidant power; DPPH = 1,1-diphenyl-2-picrylhydrazil scavenging; ORAC = oxygen radical absorbance capacity; FOS = fructooligosaccharides.

Principal component analysis (PCA) showed the distribution of the accessions studied, based on the analysis of the biochemical parameters (Figure 13). The PC1 absorbs 34.70% of the total variation, while the PC2 represents 25.92% of the total variations. The main source of variation in the data comes from the genotype (PC1) and the second source of variation comes from the plant organ, bulb, and leaf blade (PC2). The OR bulb and, to a lesser extent, the OS bulb are characterized by high glucose, sucrose, fructose, polyphenolic, and flavonoid contents, as well as high antioxidant activity, as measured by the FRAP and DPPH radical scavenging assays. In addition, the WO bulb is characterized by high total sugar content, while the ON bulb is characterized by high levels of FOS, total sugar content, ORAC antioxidant capacity, and volatiles, including organosulfur compounds, hydrocarbons, and aldehydes. The leaf blade of the studied genotypes is characterized by a correlation with volatile compounds showing high levels of ketone content, alcohols, carboxylic acids, and esters. The results demonstrated that the bulb is primarily associated with antioxidant capacity, total flavonoid content (TFC), sucrose, glucose, fructose, total sugars, and various volatile compounds such as organosulfur compounds, aldehydes, and hydrocarbons. Conversely, the leaf blade was characterized by volatile compounds such as alcohols, ketones, carboxylic acids, and esters. The results obtained delineate the differing characteristics and profiles among the studied genotypes. Furthermore, these findings suggest a specific biochemical characteristic between the leaf blades and bulbs of the onion genotypes. Overall, these results showed the antioxidant capacity, sugar profile, and volatile compound differences within the different plant organs (PO), the bulb and leaf blade, highlighting the significance of comprehensive profiling for elucidating their specific properties.

## 3. Discussion

Nowadays, consumers prefer to consume vegetables that are rich in bioactive compounds, such as polyphenols, ascorbic acid, and vitamins. Onions, being a versatile vegetable, are commonly included in the human diet as condiments or in various dishes as fresh produce. The results demonstrate that the biodiversity in onion nutritional composition is high. For the morphometric parameters, significant differences between genotypes were observed, except for leaf density. The WO showed the highest leaf blade fresh weight compared to the other genotypes. The dimensions of leaves were different among the genotypes. The WO was the best-performing genotype in terms of leaf attitude and leaf cracking. Our data confirmed previous findings, highlighting the distinctive morphology traits of the Egyptian walking onion (WO) compared to other genotypes, such as leaf and scape characteristics [28]. Aryakia et al. [29] studied the different qualitative and quantitative parameters of leaves, namely, the number of leaves, dimensions of the main leaf, leaf density, leaf attitude, and leaf cracking of *Allium* spp.

The data revealed significant interactions of the plant organ and the genotype in the biochemical profile of the onion. Numerous scientific studies have demonstrated specific beneficial properties of onions, such as antioxidant, antimicrobial, anticholesterolemic, and anticancer properties [30,31,32]. The main antioxidant compounds found in onions are flavonoids, quercetin, kaempferol, and their derivates [33]. The onion’s antioxidant activity is related to its ability to reduce the formation of free radicals and reactive oxygen species (ROS) [34,35]. The principal component analysis (PCA) showed distinct effects on antioxidant properties, sugar components, and volatile compounds in the plant organs (bulb and leaf blade) and genotypes. The bulbs were characterized by high antioxidant capacity (FRAP, DPPH, and ORAC) and flavonoid content (TFC), as well as sugars such as glucose, sucrose, fructose, and FOS. Notably, data in the literature highlighted the finding that the bulb contains the highest total phenolic content (TPC) compared to the leaf blade, suggesting that the bulb is a good source of phenolic compounds [36]. In our results, a significantly higher total phenolic content was found in the bulb, with red onion showing the highest value, followed by the Egyptian walking onion. According to our study, red onion exhibited the highest total phenolic content compared to the other onion varieties [37,38]. 

Based on the study by Yang et al. [39], which suggests variations in the phenolic content and the antioxidant capacity in relation to the different genotypes, our results provide valuable information into the profiles of bioactive compounds considering different plant organs of several genotypes. The significant interaction of PO × GE showed the genotype-specific regulation of phenolic biosynthetic pathways in response to physiological and environmental factors [40]. The total flavonoid content was determined. In line with the previous study, red onion also exhibited a high total flavonoid content [41,42].

The antioxidant capacity was measured utilizing different assays, such as FRAP, DPPH, and ORAC. Our results confirm that red onion exhibited the highest FRAP value and DPPH capacity compared to the yellow and white varieties [43,44]. The Egyptian walking onion (WO) also exhibited higher levels of antioxidant compounds. Our results align with the literature data, as the antioxidant capacity varied in relation to the genotypes, the different parts of plants, and the different methods utilized [45]. 

According to previous studies, our results showed the higher antioxidant capacity of the tested onions, suggesting that the landrace variety studied is characterized by highly nutritional properties [46,47]. The data also showed significant information on onion metabolite diversity and opportunities for targeted breeding efforts to optimize onion varieties for specific end-uses, including culinary or medicinal purposes [48]. The results obtained also confirm that the bulb serves as a reservoir of carbohydrates, particularly fructooligosaccharides, which is consistent with the literature data [49,50]. Soluble sugar content has an important role in onions, contributing to their sweetness and influencing both their taste and nutritional profile [51,52]. FOS, glucose, sucrose, and fructose are predominant. Their contents can vary significantly depending on several factors such as the cultivar, environmental conditions, plant organs, and growth stage [53,54,55]. In line with other, previous studies, our results show the different compositions of soluble sugars, highlighting significant variations between the plant organ and the genotype [56,57]. The WO bulb, in particular, showed the highest fructose content (184 g/kg d.w.). As numerous studies have shown, *Allium cepa* spp. is the main source of FOS (fructooligosaccharides). Fructans, a polysaccharide reserve found in various vegetables such as artichokes, chicory roots, onions, and garlic, are renowned for their prebiotic properties. They regulate intestinal health by selectively stimulating the growth and activity of beneficial bacteria in the colon [55,58]. Their content varies in relation to botanical variety and bulb quality [59]. ON demonstrated the highest FOS content. In line with the literature data, WO also showed a high level of FOS [60]. Additionally, OR and OS exhibited the highest amounts of glucose and sucrose, while WO showed the highest fructose content. Glucose, sucrose and fructose contribute to the sweetness of fruits and vegetables, influencing their texture and their quality traits [61,62]. Our results are in line with consumers’ preferences, which favor food items that are characterized by a distinct qualitative profile, including taste quality, soluble solid content, shapes, colors, and sweetness. Furthermore, the importance and preference of consumers for prebiotics are well recognized, with vegetables high in FOS content being particularly valued.

The results demonstrated different volatile profiles and contents among the bulbs and leaf blades and among the genotypes studied [63,64,65]. The leaf blade results were characterized by high contents of alcohols, ketones, carboxylic acids, and esters, which is consistent with previous studies indicating that the volatile profile of onions determines their flavor and aroma attributes [66,67] and holds promise for various food applications [68,69]. According to the study by Boelens et al. [70], thiosulfonates and disulfides contribute to the flavor of onions. The abundance of volatile compounds, such as organosulfur compounds, alcohols, aldehydes and esters, in both the bulb and leaf blade of onions highlights their significance when defining the overall volatile profile of onions. Our results corroborate previous studies indicating that the main volatile compounds in onions were detected by HS-GC/MS, highlighting the influence of the analytical methods used. Organic sulfides, disulfides, and trisulfides were identified as the predominant compounds found in our study, which was also reported previously by Colina Coca et al. [71].

Notably, the organosulfur compounds were the most predominant volatile compound in the bulb and leaf blade, contributing to half of the total volatile compounds, particularly for the ON bulb and WO leaf blade. This study significantly contributes to our understanding of the biological activities of onions, particularly focusing on a Sicilian landrace, WO. The study by Cecchi et al. [72] confirms our results, wherein OS and OR (*Allium cepa* L.) exhibit esters, organosulfur compounds, aldehydes, and ketones.

These findings contribute to enhancing the possibility of using these onions as ready-to-eat products, or introducing them as functional food ingredients, dietary supplements, and industrial formulations, thereby contributing to the reduction of food waste and the sustainable utilization of agricultural resources [73]. The results suggest that the bulbs of onions are rich in polyphenolic content. The Sicilian landrace, “Cipudda agghiarola” or WO, showed a significant biochemical profile, as with the antioxidant and sugar contents. This comprehensive biochemical analysis of the different genotypes of onions, particularly the Sicilian landrace WO, underscores their value as a beneficial dietary addition, offering not only a distinctive flavor but also health-promoting properties.

## 4. Materials and Methods

### 4.1. Plant Material and Morphometric Characterization

**Figure 14:**
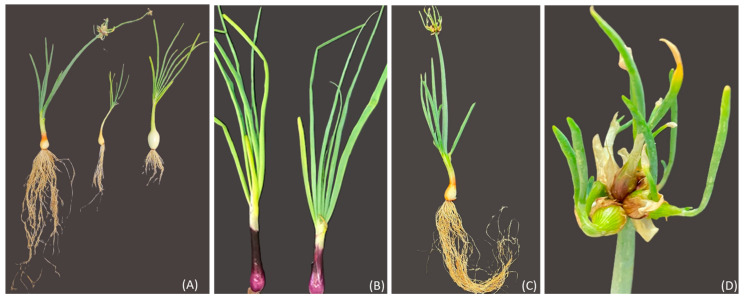
(**A**) from left to right: WO (*Allium* × *proliferum*), ON (*Allium cepa* var. *aggregatum*), and OS (*Allium cepa* var. *cepa*, cv. Stoccarda); (**B**) OR (*Allium cepa* var. *cepa*, cv. Rossa Carmen); (**C**) plant of WO (*Allium* × *proliferum*), with the vegetative stalk; (**D**) bulbils originating at the top of the plant stalk of WO (*Allium* × *proliferum*).

The perennial Sicilian landrace, “Cipudda agghiarola”, known as the Egyptian walking onion (*Allium* × *proliferum* (Moench) Schrader, WO), and the landrace “Cipudduzza” (*Allium cepa* var. *aggregatum*, ON) were compared with two commercial cultivars of onion, Stoccarda and Rossa Carmen (*Allium cepa* var. *cepa*, OS and OR, respectively) (Figure 14). The bulbs of WO and ON belonged to the active genebank of vegetables of the Department of Agriculture, Food, and Environment (Di3A) of the University of Catania, while the OR and OS bulbs were provided by the HortuSì srl seed company (Longiano, Italy). The bulbs were characterized by different sizes: 7 g for the WO, 15 g for the ON, 12 g for the OS and 13 g for the OR. The bulbs were planted on 6 September 2021 into 3.5-L pots filled with organic soil and a 2:1 ratio of agriperlite in a cold greenhouse in Catania (37°31′10.0″ N, 15°04′18.0″ E; 105 m above sea level, Sicily), under natural light and temperature conditions and following organic growing practices. After 1 month, the plants were transplanted into 16 × 16 cm pots using the same substrate as in the previous pots. The experimental design that was adopted was a randomized complete block with a single experimental factor, the genotype (GE). The experimental trial was performed with 3 replicates of 50 plants for each genotype. The plants were irrigated according to ordinary techniques; at harvest, they were collected and characterized for their main morphometric parameters, following the International Plant Genetic Resources Institute descriptors of *Allium* spp. (IPGRI): bulb fresh weight (BFW); leaf blade fresh weight (LBFW); number of leaves (NL); main leaf length (MLL); main leaf width (MLW); leaf density (LD); leaf attitude (LA); and leaf cracking (LC). The plants, after characterization, were frozen at −80 °C, freeze-dried, and ground to obtain a fine powder, and were then utilized for biochemical analysis. First, 75 mg of the freeze-dried samples was homogenized with 1.5 mL of aqueous methanol (80/20, *v*/*v*) using 2.4 mm metal beads (Omni kit 19–670, Kennesaw, GA, USA) at room temperature for 1 min at 5 m/s (OmniBeadRuptorElite, Kennesaw, GA, USA). The extracts were centrifuged at 16,000× *g* for 10 min (Centric 350, Tehtnica, Ljubljana, Slovenia) and the supernatants were transferred to a clean tube and stored at −80 °C. 

### 4.2. Biochemical Measurements

#### 4.2.1. Total Phenolic Content (TPC)

The total phenolic content (TPC) was determined by the Folin–Ciocalteu method [74]. First, 100 µL of the sample extract were mixed with 0.2 M (10% *v*/*v*) of Folin–Ciocalteu reagent; after 1 min, 100 µL of a 6% (*w*/*v*) sodium carbonate (Na_2_CO_3_) solution was added. The mixture was incubated for 60 min at 25 °C and the absorbance was measured at 750 nm using a Tecan Infinite 200 Pro M Nano+ spectrophotometer (Männedorf, Switzerland). The results were calculated utilizing a standard curve for gallic acid (y = 3.8765x + 0.0127; serial dilutions of gallic acid: 20, 40, 60, 80, and 100 µg/mL; coefficient of determination, R^2^ = 0.9998). The total phenolic content (TPC) was expressed as mg GAE/100 g d.w.

#### 4.2.2. Total Flavonoid Content (TFC)

The total flavonoid content was ascertained using the aluminum chloride colorimetric assay developed by Sembiring et al. in 2018, with some modifications [75]. First, 100 µL of the sample extract was mixed with 10 µL of 10% aluminum chloride. Subsequently, 180 µL of 80% methanol and 10 µL of 1 M sodium acetate were added. The reagent mixture was left for 40 min at 25 °C in dark conditions. The absorbance was recorded at 415 nm using a Tecan Infinite 200 Pro M Nano+ spectrophotometer (Männedorf, Switzerland). The results were determined by the standard curve of quercetin (y = 7.1021x − 0.005; serial dilutions of quercetin: 20 to 100 mg/L; coefficient of determination; R^2^ = 0.9999). The total flavonoid content was expressed as mg QE/g d.w.

#### 4.2.3. Total Antioxidant Capacity 

The antioxidant capacity was measured with a FRAP assay [76], DPPH radical scavenging activity [77], and ORAC assay [78]. For the FRAP (ferric reducing antioxidant power) assay, 100 µL of the sample extract was mixed with 200 µL of FRAP reagent. The absorbance was measured at 593 nm after 10 min at room temperature, using a Tecan Infinite 200 Pro M Nano+ spectrophotometer (Männedorf, Switzerland). The FRAP value was determined by utilizing a Trolox+ calibration curve (y = 7.0982x − 0.0363; serial dilutions of Trolox: 20, 40, 60, 80, and 100 µM; coefficient of determination, R^2^ = 0.9995). The results were expressed as µmol TE/g d.w.

For the DPPH (1,1-diphenyl-2-picrylhydrazil scavenging) assay, 100 µL of the sample extract was mixed with 200 µL of 0.1 mM DPPH radical. The absorbance was measured at 517 nm after 30 min at 25 °C with a Tecan Infinite 200 Pro M Nano+ spectrophotometer (Männedorf, Switzerland). The results were calculated using a Trolox standard curve (y = −21.0864x + 21.2202; series of Trolox dilutions: 20, 40, 60, 80, and 100 µM; coefficient of determination, R^2^ = 0.9999) and were expressed as µmol TE/g d.w.

For the ORAC (oxygen radical absorbance capacity) assay, 37.5 µL of the sample extract was mixed with 225 µL 4 µM fluorescein solution. The reaction mixture was kept at 37 °C for 30 min, and after that, 37.5 µL of AAPH solution was added. The measurement was performed using a Tecan Infinite 200 Pro M Nano+ spectrophotometer (Männedorf, Switzerland), with excitation and emission length set at 485 nm and 528 nm, respectively, for 1-minute intervals for 35 min. The results were determined by a standard curve utilizing a Trolox concentration against the average net area under the curve (AUC) for each concentration (y = 0.034140x − 0.000249; serial dilutions of Trolox: 8, 16, 24, 32, and 40 µM; coefficient of determination, R^2^ = 0.9998). The AUC value was determined as following: AUC = 0.5 + f1/f0 + … fi/f0+ … + f34/f0 + 0.5 × (f35/f0), with f0 as the initial fluorescence at 0 min and fi as fluorescence at time I. The net AUC value was calculated by subtracting the AUC of the blank from each sample and the ORAC value was expressed as µmol TE/g d.w.

#### 4.2.4. Soluble Sugar Analysis

The soluble sugar level was determined by homogenizing 150 mg of the freeze-dried samples for 1 min at 5 m/s using 2.4 mm metal beads (Omni kit 19–670, Kennesaw, GA, USA) in 3 mL of 80% methanol in water using a bead mill (Omni Bead Ruptor Elite, Kennesaw, GA, USA) [79]. The mixture was subsequently centrifuged for 5 min at 5000× *g* and the extracts were filtered through a 0.22 µm nylon filter. The analysis of sucrose, glucose, fructose, and inulin (FOS) contents was carried out utilizing an HPLC system equipped with a system controller (Shimadzu CBM-40, Kyoto, Japan), a degassing unit (Shimadzu DGU-405, Kyoto, Japan), a solvent delivery unit (Shimadzu LC-20Ai, Kyoto, Japan), an autosampler (Shimadzu SIL-20AC, Kyoto, Japan), a column oven (Shimadzu CTO-40S, Kyoto, Japan), and a refractive index detector (Shimadzu RID-20A, Kyoto, Japan). The chromatographic separation was performed by injecting 10 µL of the sample onto a 300 × 8 mm, 9 µm particle size, calcium cation exchange column (Ammerbuch, Germany) at 80 °C, with deionized water used as the mobile phase (0.6 mL/min, isocratic elution). The results were calculated with a calibration curve using serial dilutions of 0.05, 0.1, 0.5, 1.0, 5.0, 10.0, and 20.0 g/L of inulin (FOS) (y = 114.85x − 1.743; coefficient of determination, R^2^ = 0.99999), sucrose (y = 130.79x − 0.3583, coefficient of determination, R^2^ = 0.99999), glucose (y = 132.36x − 2.101, coefficient of determination, R^2^ = 0.9999), and fructose (y = 126.99x − 1.4761, coefficient of determination, R^2^ = 0.9999). The total sugar content represents the sum of the soluble sugars, and the results were expressed as g/kg d.w.

#### 4.2.5. Volatile Compounds Analysis

The analysis of the volatile compounds was performed utilizing headspace-gas chromatography/mass spectrometry (HS-GC/MS) [80]. First, 2 g of the homogenized allium sample was weighed in a 20 mL headspace vial, mixed with 6 mL of deionized water spiked with the internal standard, and immediately capped. The internal standard used was 2-octanol. Headspace extraction was carried out on an autosampler equipped with a heated agitator and a 2.5 mL heated headspace syringe (AOC6000, Shimadzu, Kyoto, Japan) with an agitator temperature of 40 °C; incubation time 45 min; headspace syringe temperature 80 °C; and volume of the sampled headspace 1 mL. The separation of the volatile compounds was performed on an Rxi 5-MS column (Restek, Bellefonte, PA, USA) by the splitless injection of 1 mL of the sampled headspace, with a helium column flow of 1 mL/min and with a temperature program as follows: hold 40 °C, 5 min; ramp to 220 °C, 10 °C/min; ramp to 300 °C, 15 °C/min; hold 300 °C, 5 min (GC2030, Shimadzu, Kyoto, Japan). The following MS parameters (TQ8040NX, Shimadzu, Kyoto, Japan) were used: ion source temperature of 280 °C; interface temperature of 300 °C; electron impact ionization; and mass scan range from 40 to 350 *m*/*z*. For each compound, Kovat’s retention index was calculated against a mix of standard alkanes, using the same temperature program. Compounds were identified using the NIST17 database. The data were normalized against the internal standard and expressed as mg/L.

### 4.3. Statistical Analysis

Statistical analysis was performed using GraphPad Prism version 8.0 (GraphPad Software, Inc., San Diego, CA, USA). A one-way analysis of variance (ANOVA) was utilized to determine variations in the morphometric traits in relation to the genotype. The significance of differences in the biochemical profile was assessed using a two-way ANOVA with Tukey’s post hoc test (*p* < 0.05) to evaluate the variation between the bulbs and leaf blades in relation to the genotype. The levels of significance are indicated as follows: * *p* < 0.05, ** *p* < 0.01, *** *p* < 0.001. Heat maps of Pearson’s correlation were generated using GraphPad Prism, and principal component analysis (PCA) was conducted using XLSTAT2018 software (Addinsoft, Paris, France).

## 5. Conclusions

The data obtained varied according to the plant organs (bulb and leaf blade) and genotypes. The developed PCA model showed higher total phenolic content (TPC) and antioxidant capacity, as measured by different assays such as DPPH, ORAC, and FRAP in the bulb compared to the leaf blade. Among the genotypes tested, the highest DPPH and FRAP activity, as well as the total flavonoid content, were observed in the bulb, particularly in Rossa Carmen (OR), followed by the Egyptian walking onion (WO). Sugar content was also found to be highest in the bulbs of all genotypes, except for glucose, which was higher in the leaf blades of WO, OR, and ON. Additionally, WO exhibited the highest content of fructose. Finally, the two landraces, ON and WO, showed the highest FOS amount. The studied genotypes also demonstrated a rich profile of volatile compounds, with alkenals, esters, and alcohols being the most predominant. The results suggest that onions are a good source of bioactive compounds, with the bulb being particularly rich in total phenolic content and sugars. This study offers valuable insights into the potential revalorization and utilization of the Egyptian walking onion (WO), which could be introduced in the form of ready-to-eat products, aligning with consumers’ preferences for its distinctive volatile profile and sweetness, thus adding value to production due to its perennial cultivation. This promotes the spread of this cultivar and enhances biodiversity.

## Figures and Tables

**Table 1:** Variation of the plant morphometric traits of the tested genotypes. Data are reported as the mean ± standard error (n = 10).

Trait	WO	ON	OS	OR	*p*-Value
BFW	19.3 ± 1.0 bc	9.3 ± 1.4 d	26.5 ± 3.1 ab	28.0 ± 1.4 a	***
LBFW	27.1 ± 1.6 a	3.7 ± 0.6 c	21.9 ± 1.7 ab	16.6 ± 1.8 b	***
NL	4.9 ± 0.3 bc	4.0 ± 0.3 c	9.0 ± 0.8 a	6.6 ± 0.2 b	***
MLL	48.1 ± 1.9 a	28.6 ± 1.2 c	43.4 ± 1.5 ab	37.8 ± 1.7 b	***
MLW	10.4 ± 0.4 a	4.1 ± 0.3 d	6.6 ± 0.5 c	6.9 ± 0.7 c	***
LD	3.6 ± 0.2	3.7 ± 0.4	4.2 ± 0.6	3.9 ± 0.3	n.s.
LA	6.8 ± 0.2 a	4.2 ± 0.2 b	4.1 ± 0.3 b	4.1 ± 0.3 b	***
LC	6.4 ± 0.2 a	4.6 ± 0.4 bc	6.3 ± 0.2 a	4.4 ± 0.3 c	***

The mean was evaluated according to the Tukey test; means that are significantly different are indicated by different letters. n.s. not significant; *** significant at *p* < 0.001; WO = Egyptian walking onion; ON = shallot; OS = Stoccarda; OR = Rossa Carmen; BFW = bulb fresh weight (g); LBFW = leaf blade fresh weight (g); NL = number of leaves (n); MLL = main leaf length (cm); MLW = main leaf width (cm); LD = leaf density (3–7); LA = leaf attitude (3–7); LC = leaf cracking (3–7).

**Table 2:** Variation of the antioxidant properties and sugar profile studied in the tested genotypes. Data are reported as the mean ± standard error (n = 3).

	TPC	TFC	FRAP	DPPH	ORAC	Total Sugars	Sucrose	Glucose	Fructose	FOS
PO										
Bulb	15.0 ± 0.5	6.1 ± 0.9	19.2 ± 3.1	38.1 ± 4.8	160 ± 13	584 ± 5	71.8 ± 8.8	57.1 ± 8.1	84.9 ± 13.5	398 ± 3
Leaf blade	12.4 ± 0.3	1.9 ± 0.3	6.8 ± 0.7	27.1 ± 1.8	123 ± 5	239 ± 6 b	33.8 ± 4.8	57.7 ± 5.5	49.4 ± 5.7	98 ± 4 b
GE										
WO	14.0 ± 0.3 ab ^1^	4.0 ± 0.6 b	12.3 ± 4.0 b	41.0 ± 4.3 a	116 ± 12 c	457 ± 8 a	28.8 ± 2.5 c	44.1 ± 6.1 c	111.7 ± 3.7 a	272 ± 5 b
ON	11.7 ± 0.4 b	1.0 ± 0.3 c	4.6 ± 0.3 c	17.6 ± 2.4 b	209 ± 5 a	471 ± 14 a	21.7 ± 4.8 d	17.2 ± 2.5 d	15.9 ± 2.3 d	416 ± 15 a
OS	12.9 ± 0.4 b	2.5 ± 0.3 c	7.7 ± 0.6 c	25.7 ± 4.8 b	141 ± 4 b	396 ± 8 b	74.0 ± 16.4 b	71.9 ± 9.2 b	55.6 ± 2.6 c	194 ± 11 c
OR	16.3 ± 1.4 a	8.5 ± 2.0 a	27.1 ± 7.0 a	46.2 ± 10.2 a	99 ± 8 d	321 ± 17 c	86.8 ± 8.3 a	96.4 ± 1.3 a	85.4 ± 3.9 b	108 ± 23 d
	Significance of the differences by the ANOVA Newman–Keuls method									
PO	**	***	***	***	***	***	***	n.s.	***	***
GE	**	***	***	***	***	***	***	***	***	***
PO × GE	***	***	***	***	***	***	***	***	***	***

PO = plant organ; GE = genotype; WO = Egyptian walking onion; ON = shallot; OS = Stoccarda; OR = Rossa Carmen; TPC = total phenolic content; TFC = total flavonoid content; FRAP = ferric reducing antioxidant power; DPPH = 1,1-diphenyl-2-picrylhydrazil scavenging; ORAC = oxygen radical absorbance capacity; FOS = fructooligosaccharides; ^1^ homogenous groups in Tukey’s post hoc test are indicated by different letters. n.s. not significant; ** *p* ≤ 0.01, and *** *p* ≤ 0.001.

**Table 3:** ANOVA of the volatile compounds in the investigated plant organs and genotypes (mg/L). The values are shown as mean ± standard error (n = 3).

	Total Organosulfur Compounds	Total Alcohols	Total Aldehydes	Total Ketones	Total Volatile Hydrocarbons	Total Carboxylic Acids	Total Esters
PO							
Bulb	20.7 ± 3.2	9.9 ± 2.1	10.3 ± 1.2	3.0 ± 0.7	2.6 ± 0.3	0.107 ± 0.018	7.8 ± 1.0
Leaf blade	18.8 ± 3.6	9.9 ± 0.6	12.1 ± 1.4	5.8 ± 0.7	2.3 ± 0.3	0.204 ± 0.023	13.4 ± 1.9
*p*-value	*	ns	*	***	ns	***	***
GE							
ON	21.1 ± 7.4 a ^1^	7.6 ± 2.1 c	15.2 ± 0.5 a	2.6 ± 0.9 c	3.4 ± 0.2 a	0.171 ± 0.043 a	9.2 ± 3.1 b
OR	21.2 ± 0.8 a	6.1 ± 1.2 c	7.2 ± 0.4 c	3.8 ± 0.7 b	1.6 ± 0.1 c	0.190 ± 0.030 a	11.4 ± 1.2 b
OS	14.2 ± 0.9 b	12.0 ± 1.0 b	10.6 ± 0.9 b	4.1 ± 1.2 b	1.9 ± 0.1 c	0.196 ± 0.019 a	14.8 ± 2.6 a
WO	22.6 ± 6.3 a	14.0 ± 2.5 a	11.8 ± 2.8 b	7.1 ± 1.0 a	2.9 ± 0.5 b	0.065 ± 0.017 b	6.9 ± 1.2 c
*p*-value	***	***	***	**	***	***	***
Bulb							
ON	37.6 ± 0.3 a	2.9 ± 0.2 d	16 ± 0.6 a	1.2 ± 0.02	3.03 ± 0.11 b	0.081 ± 0.013	2.2 ± 0.2 e
OR	20.9 ± 0.8 b	3.5 ± 0.1 d	6.7 ± 0.1 c	2.43 ± 0.40	1.48 ± 0.17 c	0.148 ± 0.011	10.1 ± 0.4 cd
OS	15.8 ± 0.5 c	14.3 ± 0.2 b	12.4 ± 0.2 b	1.47 ± 0.10	1.89 ± 0.05 c	0.171 ± 0.017	9.2 ± 0.4 d
WO	8.6 ± 1 e	19.0 ± 1.9 a	6.3 ± 0.8 c	6.71 ± 0.32	3.8 ± 0.36 a	0.028 ± 0.002	9.4 ± 0.4 d
Leaf blade							
ON	4.7 ± 0.2 f	12.4 ± 0.1 b	14.5 ± 0.6 ab	4.0 ± 1.5	3.8 ± 0.14 a	0.261 ± 0.032	16.2 ± 0.7 b
OR	21.6 ± 1.5 b	8.7 ± 0.9 c	7.7 ± 0.6 c	5.2 ± 0.4	1.62 ± 0.1 c	0.233 ± 0.050	12.7 ± 2.2 cd
OS	12.6 ± 1.3 d	9.8 ± 0.4 c	8.8 ± 0.8 c	6.6 ± 0.7	1.87 ± 0.17 c	0.220 ± 0.030	20.3 ± 1.6 a
WO	36.5 ± 0.8 a	8.9 ± 1.2 c	17.3 ± 2.7 a	7.4 ± 2.1	1.99 ± 0.44 c	0.103 ± 0.007	4.4 ± 0.6 e
*p*-value	***	***	***	ns	***	ns	***

^1^ The small letters indicate homogenous groups in Fisher’s LSD test; ns—not significant; *—*p*-value ≤ 0.05; **—*p*-value ≤ 0.01; ***—*p*-value ≤ 0.001. PO = plant organ; GE = genotype. WO = Egyptian walking onion; ON = shallot; OS = Stoccarda; OR = Rossa Carmen.

## Data Availability

Data are contained within the article and Supplementary Materials.

## Supplementary Materials

The following supporting information can be downloaded at: https://www.mdpi.com/article/10.3390/plants13131727/s1, Table S1: Volatile compounds of allium samples (Mean ± SD, n = 3).
